# A Treatise on the Nature and Cure of Night-Mare

**Published:** 1834-07-01

**Authors:** 


*Der Alp, sein Wesen und seine Heilung. Eine Monographie,* von Moritz Strahl, Dr. der Medizin, Chirurgie und Geburtshülfe, und Königlicher Kreis-Physikus.—Berlin, 1833.

*A Treatise on the Nature and Cure of Night-mare.* By Dr. Moritz Strahl.—Berlin, 1833. 8vo. pp. 253.

It is a popular opinion, that nothing qualifies a physician for treating a disease so much as the having suffered under it himself: the man who has become personally acquainted with gout or rheumatism needs no *cramming* to fix their symptoms in his memory:—" those best can paint them who have felt them most." Now, the author of the treatise before us has laboured under this painful advantage: he has been afflicted with the frightful malady which he describes, and the result has been a volume which is very creditable to him in his double capacity of a philosophic historian of the disease, and a patient investigator of its nature and symptoms.

Dr. Strahl gives the opinions of authors, from the earliest ages to the present time, on the nature and essential symptoms of incubus; and shows that most physicians have fallen into error by assuming that night-mare can only take place during sleep, and that it is always accompanied by dreams or visions; and, having demolished their theories by an appeal to facts, he proceeds to give the following definition of the disease: " Night-mare is characterized by a feeling of suffocation and oppression about the præcordia and chest, which mostly comes on during sleep; it lasts only a short time, and completely disappears after a single deep inspiration. If the attack takes place during sleep, loss of the power of speech and motion frequently occurs in addition, together with the false notion that the sense of oppression is occasioned by some foreign body." (P. 55.)

Our author remarks, that, even when digestion is perfect, various kinds of gas are developed in the alimentary canal; but, in dyspepsia attended with flatulence, much gas is disengaged, which is not only troublesome from its quantity, but injurious from its quality, and painful abdominal spasms, giddiness, fainting, &c. may arise from this source. These symptoms, however, can only be explained on the supposition that the wind (to use the popular expression) is pent up by a spasm of two different points of the alimentary canal, so that the space in which it is confined is dilated; the circulation of the blood being thus clogged, and the nerves pressed upon. That this is the fact, is shown by the removal of the unpleasant symptoms as soon as the wind is got rid of.

Now, a patient suffering from night-mare always has a weak digestion, and particularly a decided inclination to flatulence. A constriction takes place somewhere in the lower part of the alimentary canal, preventing the escape of the air downwards; the air now becomes rarefied by warmth, and the condition arises known by the name of *inflatio ventriculi.* The spasmodic attacks can easily be explained by the dilatation of a large part of the body so richly provided with nerves and blood-vessels. But this dilatation of the stomach must, in particular, prevent the free movement of the diaphragm, by pushing it upwards, and the difficulty of breathing is thus easily explained. The patients feel the necessity of taking a deep inspiration, but the attempt to do so, instead of relieving them, merely increases the distressing sensation about the præcordia: and this is quite natural; for, since the resistance of the diaphragm prevents the due expansion of the lungs, the repeated attempts at a full inspiration, instead of filling the lungs, fill the stomach. The precursory symptoms likewise admit of an easy explanation. The irritable pulse, the electric shocks, and the convulsions, arise from the tension of the nerves and the sluggishness of the circulation in the affected organs.

But, in order to constitute a complete attack of night-mare, another circumstance is wanting, namely, a dilatation of the œsophagus. Amid the morbid phenomena which have been already touched upon, the patient falls asleep. The air in the stomach continues to be rarefied by the heat, and ascends into the œsophagus, which becomes dilated, and presses on the trachea. But, if this pressure has reached its highest pitch, the patient screams out, and suddenly rises, and at the same time, by an involuntary act of swallowing, presses the air collected in the œsophagus back into the stomach, by which the distressing sense of suffocation is got rid of; or the patient may raise himself up, and discharge the air contained in the œsophagus by frequent eructations. If the patient now falls asleep again, and the spasm at the two parts of the alimentary canal is not yet perfectly resolved, the scene is repeatedly renewed, until that event takes place.

Nosology furnishes us with another disease very similar to the one we have described, namely, *globus hystericus.* In this, too, the fauces are attacked with a sense of constriction, and the patient suffers from a straitened respiration, and a sensation of pressure about the pharynx, as if a ball were fixed there. Together with this, other and frequently severe, spasmodic symptoms are present, which are usually terminated by considerable eructations. This similarity confirms the opinion which the symptoms of incubus had rendered highly probable, and shows that it is a spasmodic disease.

*Diagnosis.* This is very easy, says our author; for neither asthma, nor any variety of orthopnœa, can be confounded with this disease, if we keep in view the præcordial oppression, and the dyspnœa vanishing with the first full inspiration. Dr. Strahl would wish to change the name of the disease, and, instead of *incubus,* proposes *Inflatio ventriculi œsophagea* for the chronic form, and *Inflatio ventriculi nocturna* for an accidental attack. Dr. Strahl then shows, at considerable length, that atmospheric air may circulate in the blood-vessels; and observes, "that if we are convinced that the development of air in the body is not a pathological but a physiological process, and that it is not its formation, but its retention, which is injurious, we shall be naturally led to consider the restoration of the skin to the full use of its functions as the most pressing indication, which has not yet been done with reference to the cure of flatulence." (P. 137.)

*Prognosis.* The older writers thought night-mare a dangerous disease, while of late it has been commonly supposed not to be a disease at all, but barely a morbid symptom, and at most the effect of something which, though injurious, was but transient and unimportant; and it was imagined that it could not do any considerable injury to the system. But that night-mare is not an unimportant disease, can be testified by all who have suffered under it. A disease which returns every night, and robs several hours of their destined sleep; a disease which calls forth phenomena which accompany only the most serious disturbances of the frame, and which, by the violence of its attacks, produces and keeps up in its martyrs the fear of instant death; a disease, in short, which is complicated with so many serious anomalies of the animal functions, (or, to speak more correctly, which arises from them,) can certainly not be looked upon as unimportant.

*Treatment.* The treatment of night-mare formerly rested on no fixed principles, but was as various as the theories of the nature of the disease. The followers of the humoral pathology, who attributed incubus to a thick mucus, expected success from its removal; and it is impossible (says Dr. Strahl) to refrain from smiling at the army of remedies with which they went out to battle against the night-mare. Thus, for example, Huldenreich proposes Præparantia, incidentia, abstergentia, recludentia, purgantia universalia et partialia, discutientia, educentia, tandem viscera interna alterantia, roborantia. *(Huldenreich, Dissert. de Incub.* 1658, p. 24.*)* Wolfgang Wedel, who, as well as many other old writers, supposed plethora to be the proximate cause, recommends bleeding, cupping, setons, issues, and other depletory remedies.

Forestus, and his followers, call a whole host of nervine remedies into the lists. "Dum cubitum itur, sumatur bolus ex conservâ flor. ror. mar. lavendulæ, menthæ, pæoniæ, aut conditæ radicis pæoniæ, aut excorticatæ." *(Forest. Obs.* 52.)

The indications of cure are three:

1st. To lower the excessive sensibility of the ganglionic system;

2dly. To prevent the development of flatulence;

3dly. To excite the skin to its normal degree of activity.

But, before we enter on the consideration of these points, it will be proper, says our author, to give the treatment that should be adopted during the attack itself. At a time when, from ignorance of the nature of his disease, errors in diet, and other causes had increased the violence of his nightly attacks to a frightful pitch, Dr. Strahl had gradually accumulated a little shop-full of antispasmodics by his bedside. He tried everything from castoreum to aqua melissæ, but in vain, for his attacks frequently lasted from ten at night till three in the morning, and not a single remedy was of the slightest avail, except perhaps the aqua laurocerasi, and even that was of doubtful efficacy. He at last tried a cup of chamomile tea, and, as soon as he had swallowed it, felt a slight rumbling in the region of the pylorus, while several violent eructations demonstrated that the spasm at the cardia was also resolved. His breathing became easier, a kindly relaxation set in, and a placid sleep made our author forget his troubles. This agreeable experiment was repeated every evening, and Dr. Strahl found that he had discovered a specific against this dreadful disease. The infusion must be very weak, and drunk exceedingly hot; but hot water cannot be substituted for it. Waller, who, like our author, was for years a martyr to the disease, found equal benefit from the use of carbonic acid; and hence Dr. Strahl experienced the same effects from good bottled beer. If the chamomile tea, for any reason, cannot be administered, the next best remedy is friction of the abdomen, particularly about the region of the pylorus. Warm baths are also useful, but their temperature must not exceed 26° of Reaumur (= 90½° of Fahr.) Eating, too, is certainly beneficial, and those who go to bed with empty stomachs are most liable to attacks of night-mare.

To fulfil the first indication, *i. e.* to lower the sensibility of the ganglionic system, Dr. Strahl relies chiefly on low diet; for example, the patient's breakfast is to consist of two cups of tea, and a moderate portion of wheaten or of fine rye bread, several days old. He may eat this bread either dry, or with syrup, or with very good fresh butter. Medicines are of little or no use.

The second indication consists in curing and preventing flatulence. Dr. Strahl speaks with some approbation of a method of curing flatulence recommended by many eminent physicians from Galen onwards, namely, dry cupping; he has not, however, tried it himself. As for the long catalogue of carminatives, he thinks them beneficial in slight cases, but useless, or even injurious, in severe ones. He then proceeds to give a list of foods and drinks to be abstained from; among these he reckons "milk, buttermilk, and whey, which are extremely flatulent to persons not accustomed to their use; though they are not so to those who drink them daily, and work very hard when using them, as country people do. It was therefore a rule laid down long ago by Frederic Hoffmann, that consumptive patients can use a milk diet with advantage only when their digestive organs are in good condition, and that what is called a *whey-course* is very injurious, when prescribed for weakly townspeople, or hypochondriacs, or persons inclined to suffer from acidity or flatulence." (P. 209.)

To prevent flatulence, moreover, the bowels must be kept open, and nothing answers this purpose so well as aloes, or the mildest enemata.

The third indication is to excite the skin to a healthy degree of activity. This is to be effected by wearing a flannel shirt, by friction, and by bathing. Dr. Strahl enters at some length into the details of this last subject, but we shall not follow him, as most of our readers must be familiar with them. He defines a hot bath to be one between 30° and 40° of Reaumur, *i. e.* between 99½° and 122° of Fahrenheit! It seems that Monier, a French physician, once boiled himself for eight minutes, in a bath at 122° Fahr. and lost a pound and a half weight by the experiment. (P. 222.)

We must now conclude this faint sketch of Dr. Strahl's very elaborate work, which certainly does great credit both to his abilities and his industry. We trust, however, that he will excuse us for expressing our surprise at the strange guise in which his quotations from foreign languages appear; a surprise which is increased by the fact that the book is printed at Berlin.

